# Phytochemical profile and anti‐oxidation activity changes during ginger (*Zingiber officinale*) harvest: Baby ginger attenuates lipid accumulation and ameliorates glucose uptake in HepG2 cells

**DOI:** 10.1002/fsn3.2654

**Published:** 2021-11-14

**Authors:** Haiwen Li, Reza Rafie, Zhidong Xu, Rafat A. Siddiqui

**Affiliations:** ^1^ Food Chemistry and Nutrition Science Laboratory, Agricultural Research Station Virginia State University Petersburg Virginia USA; ^2^ Cooperate Extension College of Agriculture Virginia State University Petersburg Virginia USA; ^3^ Key Laboratory of Molecular Chemistry for Medicine of Hebei Province College of Chemical & Pharmaceutical Engineering Hebei University of Science & Technology Shijiazhuang China

**Keywords:** antioxidants, harvest time, obesity, polyphenols

## Abstract

We determined the phenolic content and anti‐oxidation properties of ginger at different harvesting time and tested its effects on lipid droplet formation and glucose uptake in HepG2 cells. Ginger samples at different stages of maturity were harvested every two weeks starting from mid‐October for 16 weeks. Our data indicate that ginger has the highest phenolic contents and superior anti‐oxidation activity when harvested early (immature baby ginger); however, the concentration of phenolic contents and its anti‐oxidation activity were progressively reduced up to 50% as ginger matures. Furthermore, the data indicate that baby ginger extract inhibits lipid accumulation and triglyceride content in oleic acid‐induced HepG2 cells up to 20% in a dose‐dependent manner. Baby ginger exhibited significant inhibition of α‐amylase enzyme activity by 29.5% and ameliorated glucose uptake in HepG2 cell at similar level. Our results suggest that harvesting ginger at an appropriate (early) time may be beneficial for optimizing its biological active contents and qualitative properties. The data also suggest that a regular use of ginger can potentially lower incidences of obesity and diabetes.

## Graphical abstract

6‐gingerol is the primary component in ginger; it is about 15‐fold and 53‐fold higher than 6‐paradol and 6‐shogaol, respectively. Consistent with TPC and antioxidant activity, the highest amount of these major phytochemicals was obtained when harvested at the first week; however, these phenolic compounds were gradually reduced to 50% of originals as ginger matured.

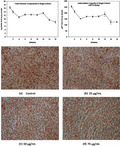

## 1 INTROCUCTION

Obesity and diabetes (especially type 2 diabetes) have been recognized as the most important public health issues worldwide. These conditions belong to a cluster of risk factors termed as “metabolic syndrome” and are closely linked as a twin epidemic. Obesity happens when the amount of calories intake exceeds the consumption. The excess in‐taken calories are converted into fat in liver and then stored as reserved fat in the adipose tissue (Deurenberg et al., 1991). Based on the report from Institute for Health Metrics and Evaluation (IHME), the global occurrence of overweight and obesity has doubled since 1980 and currently one‐third of the world's population is categorized as overweight or obese. Obesity plays a key role in metabolic syndrome and increases the risk for heart disease and other health problems. People with abnormal abdominal obesity are more likely to develop hypercholesterolemia, hypertriglyceridemia with increase in plasma low‐density lipoprotein (LDL) and decrease in high‐density lipoprotein (HDL) concentrations, which often escalate the risk of coronary heart disease, stroke, hypertension, cancer, osteoarthritis, kidney failure, gout, amputations, and blindness (Eckel et al., 2005; Isaacs & Vagnini, 2006). Obesity is a contributor to morbidity and all‐cause mortality (Hu et al., 2004; Lakka et al., 2002; Malik et al., 2004).

Type 2 diabetes mellitus, another key factor in metabolic syndrome, is defined as a group of conditions that impairs the body's ability to maintain glucose homeostasis. It is caused by the imbalance of carbohydrate, protein, and lipid metabolism, and reduction of insulin secretion from the pancreas β‐Langerhans islet cells or increase in insulin resistance from peripheral tissues (Kasuga, 2006; Lumeng et al., 2007; Zimmet et al., 2001). Worldwide prevalence of diabetes is increasing dramatically in recent decades. Based on statistics data provided by the International Diabetes Federation (IDF), approximately 463 million adults were diagnosed with diabetes in 2019, and the number are expected to be raised to 700 million by 2045. Diabetes is the major reason of hospitalization and death. It caused 4.2 million deaths and at least $760 billion dollars in health expenditure in 2019 worldwide (International Diabetes Federation (IDF) Diabetes Atlas, 2019). According to Centers for Disease Control and Prevention (CDC), diabetes is ranked 7th in the 10 leading cause of death in the United States. It causes serious long‐term complications including cardiomyopathy, nephropathy, retinopathy, renal failure, altered immune functions, oxidative stress, peripheral neuropathy, and intestinal dysfunction (Al‐Hourani & Atoum, 2007; Biessels et al., 2004; Martinez‐Tellez et al., 2005).

Obesity and diabetes can be managed by various treatment options. However, severe side effects has been cautioned with the lifelong medication. For example, thiazolidinediones (TZDs), the first‐line prescription medicine to reduce insulin resistance, have been reported with the side effect of weight gain, pedal edema, fluid retention, and congestive heart failure (CHF) (Nesto et al., 2003). Plant‐based natural products are used as complementary therapy or alongside anti‐diabetes medications (McMacken & Shah, 2017; Pandey et al., 2021). Perennial herb ginger (Zingiber officinale Roscoe, Zingiberaceae) has been widely used for centuries in ancient China and India as a folk medicine (Tabatabaei‐Malazy & Larijani, 2015). Extensive studies of ginger demonstrated ginger's diverse beneficial health effects including lipid lowering, anti‐obesity, anti‐diabetic, anti‐cancer, anti‐inflammation, anti‐oxidation, gastro‐intestinal tract protection, and micronutrients absorption stimulant (Butt & Sultan, 2011; Grzanna et al., 2005; Islam & Choi, 2008; Jayarathne et al., 2017; Jiang et al., 2006; Mahmoud & Elnour, 2013; Ojewole, 2006; Srinivasan, 2017; Suk et al., 2016; Wang et al., 2017). Aqueous ginger extract also revealed anti‐obesity effects when supplemented with a high‐fat diet in a mice feeding experiment; the data analysis suggested that the active compounds in ginger may partially inhibit the intestinal absorption of dietary fat by blocking its hydrolysis and ultimately reducing the elevation of the plasma triacylglycerol levels (Han et al., 2005). Ginger also showed potential functions in prevention of obesity through the alleviation of inflammation. When Sprague‐Dawley rats were fed the high‐fat diet with ginger extract, body weight gain and total weight of white adipose tissue mass were significantly decreased and serum and liver lipids were also reduced (Kim et al., 2018). Ginger extracts suppressed the expression of genes related to adipogenesis and pro‐inflammatory cytokines, such as peroxisome proliferator‐activated receptor γ (PPAR‐γ) and adipocyte protein 2 (aP2) (for adipogenesis) and TNF‐α, interleukin‐6 (IL‐6) and MCP‐1 (for pro‐inflammatory cytokines). Anti‐diabetic effects of ginger have been demonstrated in an animal model. When rats were fed a high‐fat diet with ginger extracts, serum insulin concentration was significantly ameliorated and glucose tolerance was increased (Jayarathne et al., 2017; Srinivasan, 2017). Ginger also has a potential effect in decreasing the risk of some chronic complications caused by diabetes. A randomized, double‐blind clinical trial study showed that ginger extracts significantly improved the serum levels of fasting blood sugar, hemoglobin A1c, apolipoprotein B, apolipoprotein B/apolipoprotein A‐I, and malondialdehyde in type 2 diabetic patients (Khandouzi et al., 2015). Furthermore, ginger has been reported to prevent obesity and attenuate the complications by suppressing inflammatory responses of adipose tissue (Grzanna et al., 2005; Misawa et al., 2015).

The concentration of primary polyphenolic components in ginger such as gingerols, shogaols, zingerone, and paradols was fully investigated (Saravanan et al., 2014; Tzeng & Liu, 2013). Gingerols are the major aromatic and pungent compounds present in ginger. Its anti‐inflammatory properties have been explored both under in vivo and in vitro conditions. Particularly, PPAR‐δ signaling pathway in adipocytes has been shown to be regulated by 6‐gingerol and 6‐shogaol (Misawa et al., 2015), and enhanced PPAR‐δ‐dependent genes expression was found in cultured human skeletal muscle myotubes (Suk et al., 2016). Furthermore, the effects of 6‐gingerol on diabetes were investigated with a Lepr^db/db^ type 2 diabetic mice model (Samad et al., 2017). It was found that 6‐gingerol played a key role in glucose homeostasis via amelioration of glucose‐stimulated insulin secretion and dramatically improving glucose tolerance through increasing the expression of Glut 4 glucose transporters.

In the present study, we examined phytochemical profile and health benefits of the “immature” baby ginger and compared this with “fully mature” product. Total phenolic content and anti‐oxidation properties of ginger during the 4‐month harvesting period were evaluated. Anti‐obesity effects of baby and matured ginger were also investigated using HepG2 human hepatic cells model. In addition, the potential anti‐diabetic properties of ginger extracts on in vitro α‐glucosidase activity and glucose uptake in HepG2 human hepatic cells were further examined.

## 2 MATERIALS AND METHODS

### 2.1 Materials

2,2‐Diphenyl‐1‐picrylhydrazyl radical (DPPH), Folin–Ciocalteu reagent, gallic acid, and 6‐hydroxy‐2,5,7, 8‐tetramethylchroman‐2‐carboxylic acid (Trolox), Roche Cell Proliferation Reagent WST‐1, 6‐gingerol, 6‐shogaol, and 6‐paradol were purchased from Sigma‐Aldrich. Human hepatic cell HepG2 was purchased from ATCC (American Type Culture Collection). Culture medium Eagle's Minimum Essential Medium (EMEM) with 1% antibiotic/antimycotic, 10% fetal bovine serum (FBS), 0.25% trypsin, and 1 mM EDTA were purchased from Invitrogen. Oil Red O staining kit was purchased from Lifeline Cell Technology. Triglyceride Colorimetric Assay kit and Glucose Uptake Cell‐Based Assay Kit were purchased from Cayman Chemicals.

### 2.2 Ginger cultivation and methanol extraction

Chinese ginger roots were planted in one gallon pots in a greenhouse using seed rhizome in 2017. After 4 months of greenhouse cultivation, ginger plants were transplanted into high tunnel. Pest and disease management and mounding were applied regularly. Ginger root samples from three individual plants were collected (8 months after sprouting) biweekly from October 2017 for 16 weeks. Samples were cleaned with DI water and diced into small pieces and then freeze‐dried under low pressure (200 mTorr) in a freeze drier (SP Scientific). Dried samples were milled using a Scienceware Bel ArtMi‐cromill (Pequannock, NJ) and then passed through a 40 mesh size (size in 400 µm). The ginger powder was extracted with 80% methanol (50 mg/ml) for 18 h at ambient temperature with 250 rpm on a Scilogex SK‐O330‐PRO orbital shaker (Scilogex, CT) followed by centrifugation at 2,500 × *g* 20°C for 40 min. The supernatant was directly used to evaluate antioxidant capacity. For all cell‐based experiments, the methanol extracts were evaporated in a nitrogen evaporator (Organomation Associates, Inc.) followed by freeze‐drying overnight to remove the traces of methanol/water residues. The dried residues were dissolved in DMSO. All extracts were kept under nitrogen and stored at −20°C until further analyzed.

### 2.3 Total phenolic content

TPC was measured using Folin & Ciocalteu's (FC) reagent (Li & Parry, 2011) with slight modification to adopt a 96‐well microplate version. Briefly, the reaction was conducted in a mixture containing 80 μl pure water, 20 μl ginger extract (25 mg/ml), 20 μl FC reagent, and 160 μl 7% sodium carbonate. The reaction was kept in the dark for 2 h at ambient temperature. Absorbance was measured at 765 nm using a microplate reader SpectraMax M5 (Molecular Devices, LLC.). Total phenolic content was determined using gallic acid as a standard curve. The analysis was performed in triplicate, and data were expressed as milligrams of gallic acid equivalents (GAE)/g of dried sample.

### 2.4 Antioxidants assay

#### 2.4.1 DPPH assay

2, 2‐diphenyl‐1‐picryhydrazyl (DPPH^•+)^ free radical was generated by dissolving DPPH in 80% methanol to final concentration of 0.2 mM. Scavenging capacities of the ginger extracts were assayed using a procedure as previously described (Li & Parry, 2011). 100 µl of freshly prepared DPPH^•+^ methanol solution was mixed with 100 µl of ginger extract (0.5 mg/ml) in a 96‐well microplate. Absorbance readings were measured at 517 nm after a 30‐min reaction. 80% methanol was used as a blank. Trolox was used to generate a calibration curve (0–70µM). DPPH^•+^ scavenging capacity of ginger extracts was demonstrated by plotting against Trolox antioxidant standard curve. The experiment was conducted in triplicate, and data were expressed as µM of Trolox equivalents (TE)/g of dried sample.

#### 2.4.2 ABTS assay

2,2′‐azino‐bis(3‐ethylbenzothiazoline‐6‐sulfonic acid (ABTS^•+)^ scavenging capacities of the ginger extracts were evaluated following a previously described procedure with slight modification (Li & Parry, 2011). Initially, 5 mM highly oxidized ABTS + cation radical was generated by thoroughly mixing ABTS and manganese dioxide at ambient temperature for 18 h. Extra manganese dioxide was removed after reaction by filtration through Whitman paper. The ABTS^•+^ solution was then diluted with 0.1 M phosphate buffer saline (pH 7.4) to reach an absorbance of around 0.5 at 734 nm. For ABTS assay, 230 µl ABTS^•+^ solution was added to a 96‐well microplate and absorbance was recorded at 734 nm. 20 μl of standard or ginger extracts (1 mg/ml) was then added and followed for 30 s by intermittent shaking at room temperature. The absorbance was measured again at 734 nm. 80% methanol was used as the solvent blank. Differences between ABTS reading before and after adding standard or sample were calculated and ABTS+reducing activity in ginger extracts was determined against a Trolox standard curve. The assays were performed in triplicate. Trolox equivalents (TE) were calculated by expressing in µM TE/g dried sample.

### 2.5 Chemical composition of ginger extracts by HPLC

Key phytochemicals from ginger extracts (6‐gingerol, 6‐paradol, and 6‐shogaol) were quantified by HPLC. Dried ginger powder (100 mg) was accurately weighted and mixed with 3.0 ml of ethanol. The mixture was vortexed and stored at 4°C for 24 h. The resulted solution was centrifuged at 12,281 *g*, and then 500 µl of top clear solution was transferred to a sample vial and diluted with 500 µl of acetonitrile for analysis using a HPLC system (Thermo UltiMate 3000). A 20 µl of sample was injected on a C18 Agilent 5TC‐C18 250 × 4.6 mm using a mobile phase consisting of acetonitrile‐H_2_O (50:50) for a 50 min run. The separation of samples was detected using a UV detector at 280 nm. The components of the ginger were identified by comparing the retention time with the corresponding reference standard. The authentic standards of 6‐gingerol (98%, Sigma‐Aldrich), 6‐shogaol (98%, Sigma‐Aldrich), and 6‐paradol (98%, Sigma‐Aldrich) were used and the concentration (mg/g) of the components was calculated using a calibration curves for each standard compound (20 µ g/ml 1,000 µ g/ml) and data are expressed as mg/g of ginger powder. Each sample was tested in triplicate independently.

### 2.6 Hepatic cell culture and cytotoxicity assay with cell viability

Human HepG2 cells were propagated in T‐75 flasks with Eagle's Minimum Essential Medium (EMEM) supplemented with 1% antibiotic/antimycotic solution and 10% fetal bovine serum (FBS). Cells were incubated at 37°C in a humidified atmosphere with 5% CO_2_. Cytotoxicity assay was conducted with WST‐1 following the manufacturer protocol (Roche Applied Science, Germany). For cytotoxicity, 1 × 10^4^ cells were seeded in 96‐well plates and cultured as described above. Cells were then treated with 0, 25, 50, 75,100, 150, 200, and 300 µg/ml of baby (week 1) or mature (week 16) ginger extracts (dissolved in DMSO) followed by incubation at 37°C for 24 h. The cells were then treated by adding 10 μl WST‐1 and incubated at 37°C for additional 2 h prior to the absorbance measurement at 440 nm using the microplate reader (SpectraMax M5). All measurements were recorded in triplicate.

### 2.7 Lipid accumulation in oleic acid‐treated HepG2 cells

Lipid accumulation model was established by treating the human HepG2 cells with an oleic acid: BSA complex solution (at molar ratio of 6:1) according to the previously described method. HepG2 cells were cultured in EMEM supplemented with 1% antibiotic/antimycotic solution and 10% fetal bovine serum (FBS) in 96‐well microplate. 60% confluent cells were pretreated with 0, 25, 50, and 75 µg/ml of baby ginger (week 1) or mature ginger (week 16) extracts in growth medium for 24 h. After pretreatment, the cells were cultured for another 24 h with new medium supplemented with fresh ginger extract and 200 µM oleic acid. Lipid accumulation was determined by staining with Oil Red O stain.

### 2.8 Oil Red O staining

Lipid droplets accumulation in human HepG2 cells was detected by Oil Red O staining according to the manufacturer manual (Lifeline Cell Technology). HepG2 cells were incubated for 24 h with week 1 or week 16 ginger extracts co‐cultured with 200 µM oleic acid. Cells were washed twice with ice‐cold PBS (pH 6.8) for five minutes and fixed with 4% paraformaldehyde fixative solution for 20 min. After washing 2 times with distilled water, cells were treated with 100% 1, 2‐propanediol dehydration solution for 10 min followed by 37°C incubation for 30 min in Oil Red O stain solution. Cells were then treated with 85% 1, 2‐propanediol stain differential solution for 1 min and then washed with distilled water several times until the water contains no visible pink color. After Oil Red O stain, microscope images were recorded to observe pink oil droplets staining in HepG2 cells. Cells were photographed using a phase‐contrast microscope (Nikon) in combination with a digital camera at 20× magnification. For quantitative measurement, the stained cells were dried completely and isopropyl alcohol was added to each well and gently mixed for 15–30 min. The absorbance at 490 nm was then recorded using a microplate reader (SpectraMax M5). All experiments were repeated 6 times.

### 2.9 Triglyceride content assay

HepG2 hepatic cells were plated in 6‐well plates at a density of 2 × 10^6^ cells per well and incubated for 24 h as described above. Thereafter, cells were treated with 0, 25, 50, and 75 µg/ml of ginger extracts. Cells were incubated at 37°C with 5% humidified CO_2_ for another 24 h. Ginger‐extract‐pretreated cells were fed with fresh ginger extracts in growth medium supplemented with oleic acid (200 µM) and then incubated for another 24 h under similar condition. Cells were then harvested and subjected to Cayman Chemical triglyceride colorimetric assay following the manufacture's protocol (Ann Arbor, MI). In brief, cells were carefully washed twice with ice‐cold PBS and collected with a rubber cell scraper. Cells were then spin down by centrifugation at 2,000 *g* for 10 min at 4°C and re‐suspended in 500 μl ice‐cold diluted Standard Diluent Buffer. After sonication, cell suspension was collected by centrifugation at 2,000 *g* for 10 min at 4°C. 10 μl of supernatant or positive control was added to each well and mixed with 150 μl diluted enzyme mixture solution containing lipoprotein lipase, glycerol kinase, glycerol phosphate oxidase, and peroxidase. Reactions were incubated for 15 min at room temperature, and OD was measured at 550 nm using a microplate reader SpectraMax M5. Triglyceride content (% of control) was expressed as a ratio of the triglyceride content with ginger treatment relative to the non‐treated control (*n* = 5).

### 2.10 In vitro α‐amylase inhibition assay

Alpha‐amylase, type VI‐B from porcine pancreas (Sigma‐Aldrich Chemical Co.) was dissolved in amylase buffer (phosphate buffer with 100 mM potassium chloride and 60 mM calcium chloride, pH 6.9) at a concentration of 0.5 U/ml. 2‐chloro‐p‐nitrophenyl‐α‐D‐maltotrioside (CNPG_3_) (Sigma‐Aldrich Chemical Co.) was used as the substrate, and acarbose (0.1 mg/ml) was used as positive control for the α‐amylase activity assay. Ginger extract (0.1 mg/ml) from week 1 and week 16 samples was examined. An inhibition assay was conducted according to the protocol described by Gella et al. (1997) with a minor modification. In brief, the mixture of 60 μl control/sample solution with 20 μl enzyme solution (0.5 U/ml) was first incubated for 10 min at 37°C. After incubation, 150 μl of 2.5 mM CNPG_3_ solution in amylase buffer was added, and the reaction was then incubated for additional 45 min at 37°C. The α‐amylase activity was quantified by measuring the 2‐chloro‐4‐nitrophenol release from CNPG_3_ at 405 nm with the microplate reader. The amylase buffer (pH 6.9) was used as control. All measurements were recorded in triplicate. The inhibition of α‐amylase activity was expressed as percentage of the control.

### 2.11 Glucose uptake assay using 2‐NBDG in human HepG2 cells

Glucose uptake in the presence or absence of ginger extracts was conducted using a Cayman Chemical Glucose Uptake Cell‐Based Assay Kit following the manufacture's protocol (Ann Arbor, MI). Fluorescently labeled deoxyglucose analog 2‐NBDG was used for the direct detection of glucose taken up in cells, and the fluorescence was quantified with the microplate reader. In brief, HepG2 hepatic cells were cultured in 96‐well plates at a density of 1 x 10^4^ cells per well in EMEM supplement with 1% antibiotic/antimycotic solution and 10% fetal bovine serum (FBS). Cells were incubated at 37°C with 5% humidified CO2 overnight. Ginger extracts (baby ginger or mature ginger) at concentrations of 0, 25, 50, and 75 µg/ml were then added to the cells and then incubated for an additional day. The cell culture medium was then changed to glucose‐free DMEM culture medium with or without ginger extracts and cultured for 6 h. Thereafter, 2‐NBDG was added to a final concentration of 200 µg/ml. Cells were co‐cultured with ginger extracts and 2‐NBDG for another 16 h. At the end of the treatment, plate was centrifuged for 5 min at 400 *g* at room temperature and supernatant was aspirated. Cells were washed twice with 200 µl of Cell‐based Assay Buffer. Fluorescence was detected with plate reader (SpectraMax M5, Molecular devices) under excitation/emission of 485/650 nm. All experiments were repeated 5 times.

### 2.12 Statistical analysis

Tests were conducted with data reported as mean ± standard deviation (*n* = 3 or 5). Analysis of variance (one‐way ANOVA) and Tukey's HSD post hoc analyses using Sigma Plot Statistics13 software were used to determine differences among group means. Statistical significance was defined as *p* < .05 and indicated by “*”; *p* < .01 and indicated by “**”.

## 3 RESULTS AND DISCUSSION

### 3.1 Effect of harvest time on the total phenolic content

**FIGURE 1:**
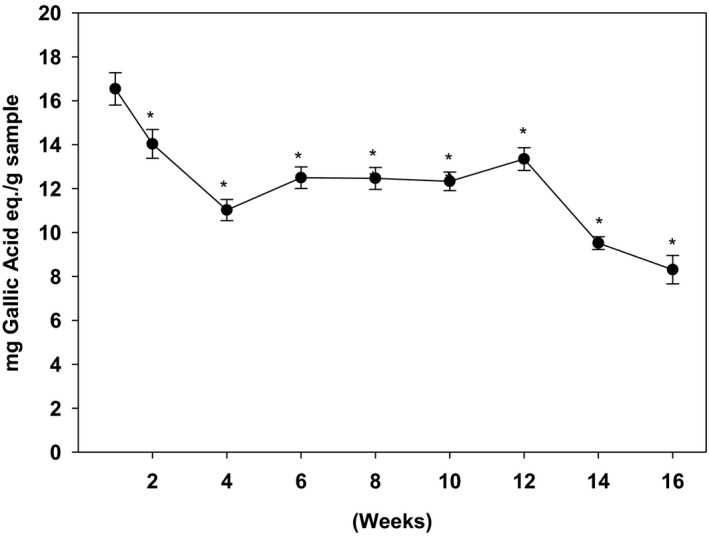
Effect of harvesting time on the Total Phenolic Content (TPC) of ginger. The TPC was determined using Folin & Ciocalteu's (FC) reagent as described in the text. Data is expressed as mean ± *SD* for at least 3 experiments. The data was analyzed by one way ANOVA with Tukey's HSD post hoc test. * represents a significant difference at *p* < .05 when compared to week 1 samples

As shown in Figure 1, the phenolic content was the highest in the week 1 harvested ginger samples, which gradually declined during the following weeks. Baby ginger (week 1) had the highest amount of TPC (16.9 mg GAE/g dry powder). During the first month of harvest, the TPC drastically declined to 10.9 mg GAE/g dry powder (week 4). The amount of TPC was then stabilized during the second and third month (week 6–12). Continual reduction of TPC was observed during the last month of cultivation (week 14–16). By the end of the harvest period at week 16, TPC of matured ginger dropped 50% compared with baby ginger at week 1.

### 3.2 Effect of harvest time on anti‐oxidation activity

**FIGURE 2:**
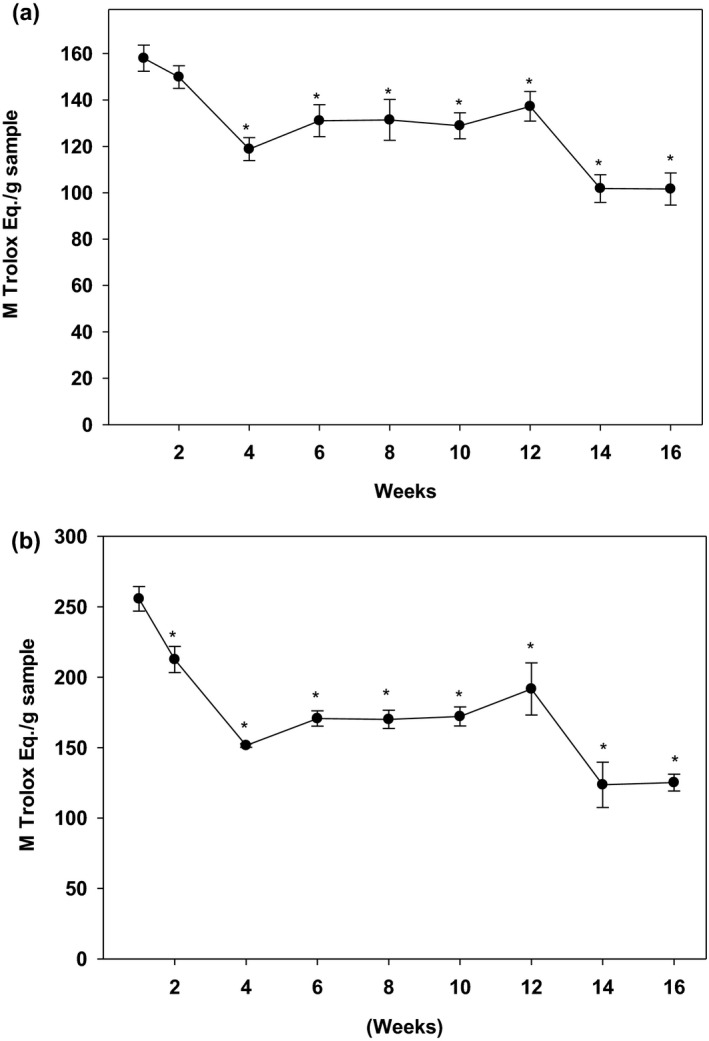
Antioxidant properties of ginger extracts. The antioxidation activity in ginger samples were determined using (a) DPPH‐ and (b) ABTS‐ free radical scavenging capacity assays as described in the text. Data is expressed as mean ± *SD* for at least 3 experiments. The data were analyzed by one way ANOVA with Tukey's HSD post hoc test. An “*” represents a significant difference at *p* < .05 when compared to week 1 sample

Results, as shown in Figure 2a,b, indicated that both DPPH and ABTS scavenging activities followed a similar pattern to that of TPC. The baby ginger exhibited the highest levels of DPPH free radical scavenging capacity at week 1 (135.3 TE µM/g dry sample), which decreased and stabilized at about 100 TE µM /g dry sample during the following 12 weeks. Similar to TPC, the DPPH activity was drastically reduced again during the last 4 weeks of harvest. A similar result was obtained for the ABTS+• scavenging activity with a highest ABTS value found in the first week (255.6 TE µM/g dry sample) which reduced significantly during the following 16‐week period. After reaching to a steady plateau stage for 2 months, matured ginger lost approximately 41% anti‐oxidation capacity when it was harvested at the 16th week. These results demonstrated that baby ginger has highest anti‐oxidation capacities, and antioxidant activity declined as ginger becomes mature.

Phytochemicals are widely distributed in plant products. The health‐promoting effects of those compounds, such as high antioxidant capacity and free radical scavenging ability, attracted special attention for decades (Kahkonen et al., 2001). Natural antioxidants, as an unconventional therapy with less side effects and high potency, are used to combat numerous health problems, which fascinated the food and pharmaceutical industries (Yeh et al., 2014). Ginger has been considered as a potential agent in prevention and treatment of many oxidative stress‐related diseases, including obesity and diabetes (Han et al., 2005; Kim et al., 2018; Suk et al., 2016). The present study investigated ginger for its total phenolic content, anti‐oxidation activity, and the key polyphenolic compounds at different maturity stages during the 4‐month harvesting period. Differences between baby (young) and matured gingers for their abilities to affect lipid accumulation, glucose uptake in HepG2 hepatic cells, and in vitro α‐amylase inhibition were also examined. Compared with the matured ginger, the major product being sold in markets and wildly used as a spice, the immature baby ginger is juicier and plump, with a creamy white bulb and vibrantly pink colored. Baby ginger is more perishable, less pungent, and fibrous compared with the matured ginger. However, the phytochemical profile and health benefits of the baby ginger compared with the fully matured ginger are not widely known. It is also not known if the harvesting time has any effect on the ginger quality and its advantageous effects on human health. To our best knowledge, the present study is the first to reveal the dynamic changes of the total phenolic contents, anti‐oxidation properties, key phytochemicals, and biological activities during early or late harvesting. These observations were not surprising because several previous studies have shown that the polyphenol contents can vary when an immature fruit ripens to mature fruits and undergoes biochemical changes in their composition, which also depends upon the environmental and the genetic factors. For example, studies have shown the phenolic contents were higher in the unripen fruits (Elmastaş et al., 2017; Tulipani et al., 2011) and their levels decreased on maturity in grapes (Kennedy et al., 2000), golden Himalayan gooseberry (Belwal et al., 2019), white mulberries (Belwal et al., 2019), bush cherry (Fu et al., 2021), olives (Vildan, 2015), and peaches (Belhadj et al., 2016). Fruits accumulate polyphenols when they are unripen to protect them from fruit‐borne diseases during the process of maturity (Lattanzio et al., 2008; Prusky & Keen, 1993). Reports suggest that phenolic contents of fruits are oxidized or transformed to pigments during ripening process (Catoni et al., 2008; Schaefer et al., 2008). Immature or baby ginger typically has a thin white coat which is changed to a thick dark‐brown coat on maturation. It is possible that a decline in polyphenolic content during progression from baby ginger to mature ginger is due to their oxidation and transformation into brown color skin synthesis. Further studies are required to identify these compounds. Interestingly, our data are consistent with earlier findings on different varieties. For example, Ghasemzadeh reported that the total phenolic content for two varieties of young ginger (*Halia Bentong* and *Halia Bara*) with methanol extraction (Ghasemzadeh et al., 2011) was 10.1 and 13.4 gallic acid equivalent mg/g dry powder, respectively. However, TPC in matured ginger was found to be 4.31 mg gallic acid equivalent/g sample (Tanweer et al., 2020). In our present study, baby ginger (week 1) extract has shown the highest amount of TPC (16.9 mg gallic acid equivalent mg/g dry powder) and TPC was dramatically dropped to 50% when they were harvested after 4 months. Anti‐oxidation property changes measured by DPPH and ABTS methods have also demonstrated a similar pattern. These data suggest that consumption of young ginger, harvested at an early stage, provides more nutritive bioactive compounds for health benefits than those harvested at a mature stage.

### 3.3 Effect of harvest time on the key phytochemicals

**FIGURE 3:**
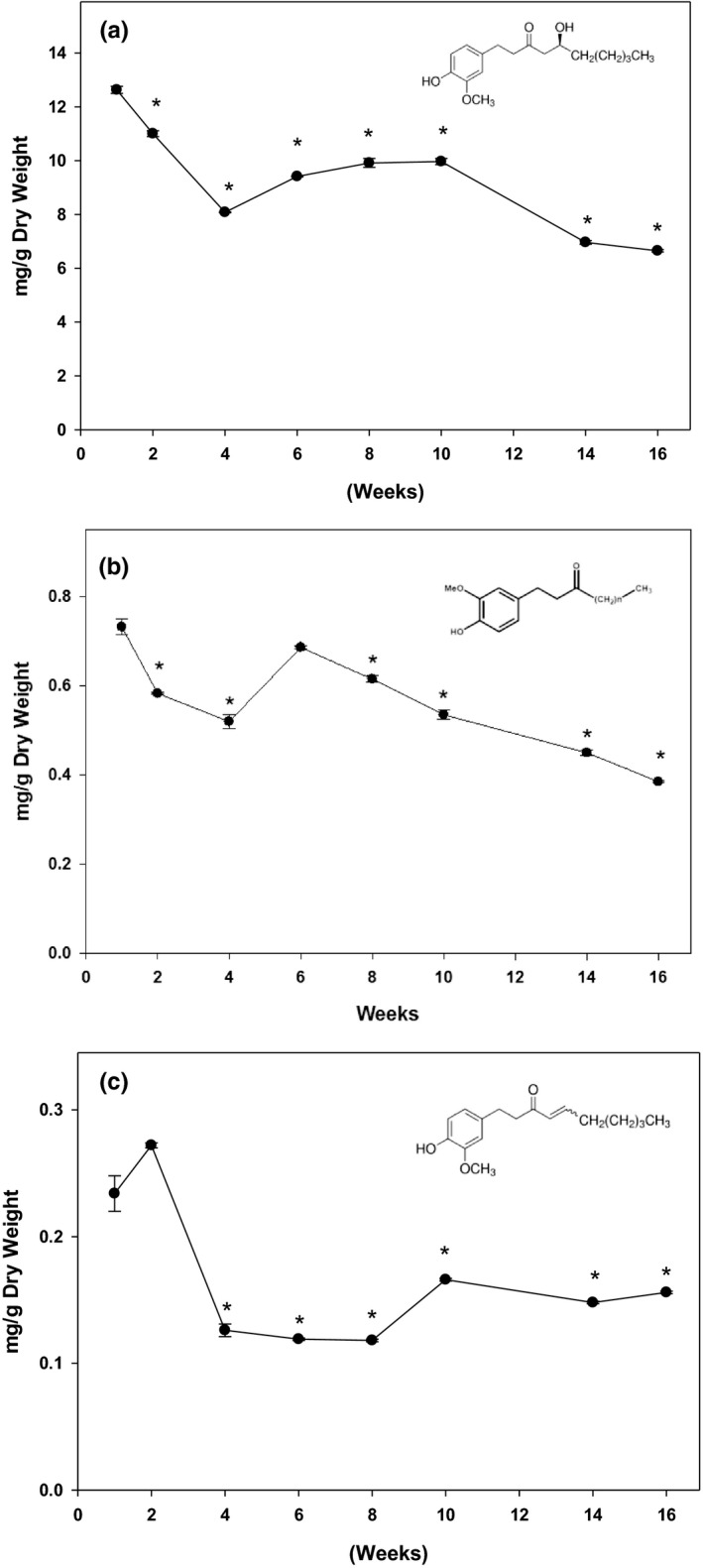
Effect of Harvest time on the key phytochemicals. The key phytochemicals in ginger samples were quantified for (a) 6‐gingerol, (b) 6‐paradol, and (c) 6‐shogaol using a HPLC system. Individual phenolic compounds were identified using authentic external standards and are expressed as mg/g dry weight. Data are expressed as mean ± *SD* for at least 3 samples. The data were analyzed by one way ANOVA with Tukey's HSD post hoc test. * represents a significant difference at *p* < .05 when compared to week 1 samples

Ginger samples at different maturity stages were investigated for variation in their key phytochemicals concentrations. Individual phenolic compounds including 6‐gingerol, 6‐paradol, and 6‐shogaol were quantified, and the results are shown in Figure 3. Overall, 6‐gingerol is the primary component in ginger; it is about the 15‐fold and 53‐fold higher than 6‐paradol and 6‐shogaol, respectively. Consistent with TPC and antioxidant activity, the highest amount of these major phytochemicals was obtained when harvested at the first week; however, these phenolic compounds were gradually reduced to 50% of originals as ginger matured.

To understand the mechanism underlying the anti‐obesity and anti‐diabetic properties of ginger, we quantified the key phytochemicals at different mature stages in Chinese ginger. Gingerols are the major aromatic and pungent compounds well defined in ginger (Semwal et al., 2015). 6‐gingerol is the major constituent of the gingerols along with 6‐shogaol and 6‐paradol. They contribute the major valuable health effects in ginger products. In our present study, individual phenolic compounds were quantified during 4‐month harvesting period. Consistent with the literatures, 6‐gingerol is the most abundant component in Chinese ginger. The amount of 6‐gingerol was 15‐fold and 53‐fold higher than that of 6‐paradol and 6‐shogaol, respectively. The highest amount of 6‐gingerol was overserved in baby ginger, and the amount was gradually reduced to 50% as ginger matured at 16th week. Although statistically non‐significant, a trend in increase in total polyphenol (Figure 1), anti‐oxidation activity (Figure 2), and key polyphenols (Figure 3) at week 12 is evident. This could possibly be resulted due to the change in environmental temperature and/or moisture content, which are difficult to control; however, there are distinct differences between these parameters between baby and mature ginger. Furthermore, the data point at week 12 was missing from data in Figure 3 due to loss in sample collection because of unavoidable Christmas holidays; however, the general trend is consistent with data in Figures 1 and 2.

### 3.4 Effects of ginger extract on HepG2 cytotoxicity

**FIGURE 4:**
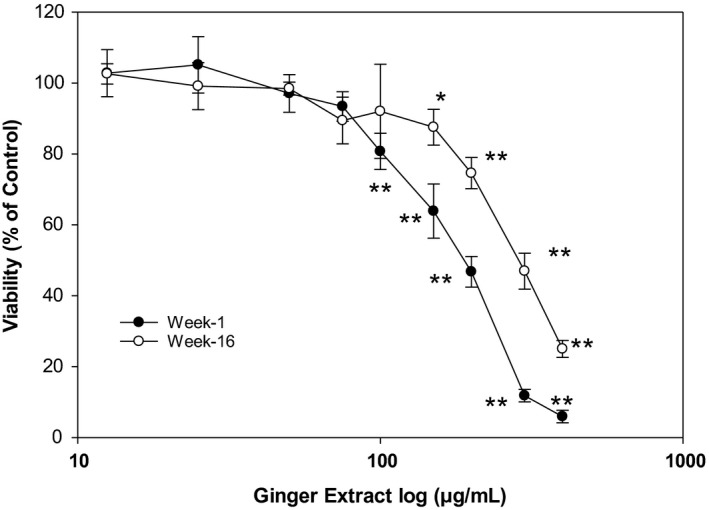
Effect of ginger on cellular cytotoxicity in HepG2 cells. HepG2 cells were treated with varying concentrations of ginger extract (0, 25, 50, 75, 100, 150, 200, 300, and 400 μg/ml) for 24 h before cell viability was examined. Cell viability was determined using a WST‐1 assay as described in the text. Data as mean ± *SD* for at least 3 experiments are plotted on log scale for X axis and analyzed using one‐way ANOVA with Tukey's HSD post hoc test. * or ** represents a significant difference at *p* < .05 or *p* < .01, respectively when compared to control (0 g/ml)

As shown in Figure 4, no statistically significant inhibition in cell viability was observed when the hepatic cells were treated with 75 µg/ml or lower ginger extracts when compared to that of the control group. In contrast, significant cytotoxicity (*p* < .01) was observed when the cells were treated with 100 µg/ml or higher concentration of ginger extracts. The calculated IC_50_ was found to be 186 µg/ml in week 1 ginger extract, and it is significantly lower than in week 16 ginger extract (278 µg/ml). These results indicate that ginger extracts up to 75 µg/ml did not cause cellular cytotoxicity in HepG2 cell. Therefore, further cell‐based experiments were conducted with ginger extracts concentration at 75 µg/ml. Higher concentration of ginger over 75 µg/ml was cytotoxic to hepatic cancer cells. In the present study, we did not investigate the cellular process involved in the cytotoxic effects of higher dose of ginger on liver cancer. However, it is likely be through extrinsic and/or intrinsic apoptotic pathways.

### 3.5 Baby ginger extract inhibits lipid accumulation in oleic acid‐treated HepG2 cells

**FIGURE 5:**
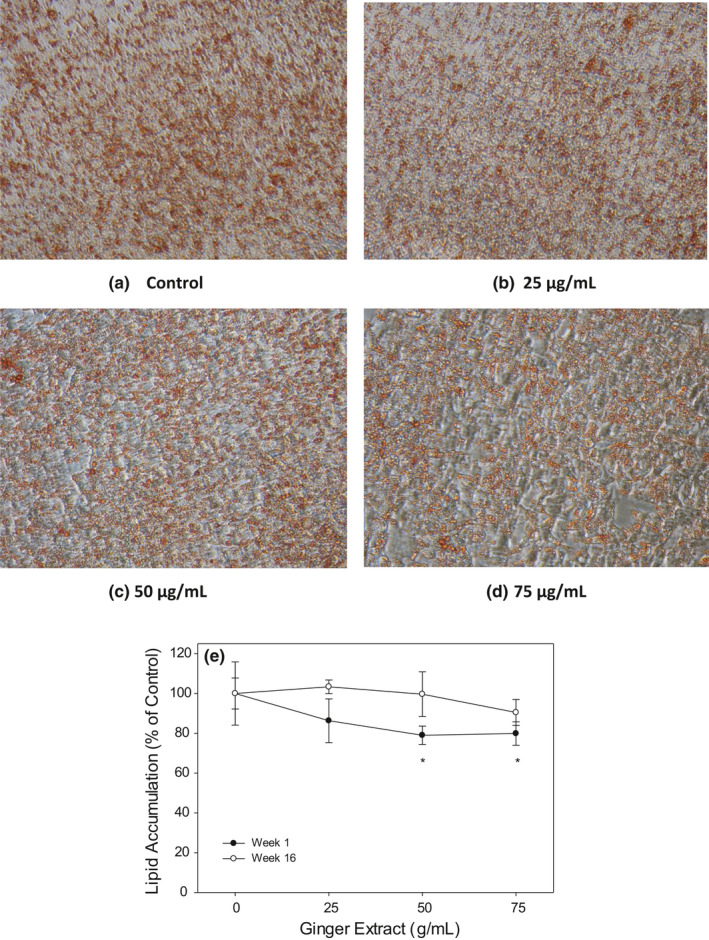
Effect of ginger extract on fat accumulation in oleic acid treated HepG2 cells. Human HepG2 cells were treated with the 200 μg/ml oleic acid with ginger extracts (baby or mature ginger) at 0, 25, 50 and 75 μg/ml. The effects of ginger extracts on fat accumulations was evaluated microscopically by staining with Oil Red‐O (a–d). The stained oil droplets were also extracted and quantified spectrophotometric ally (e). The results represent mean ± *SD* (*n* = 5). *: *p* < .05 compared to 0 g/ml

To investigate the effects of ginger extracts on attenuation of intracellular lipid accumulation, HepG2 cells were treated with oleic acid to accelerate the oil droplet formation. Baby ginger (week 1) and mature ginger (week 16) were used to study the effects of ginger on inhibiting lipid accumulation through Oil Red O staining method. As shown in Figure 5a–d, lower concentration of week 1 ginger extracts (25 µg/ml) did not show much inhibition on intracellular lipid droplet accumulation in HepG2 cells compared to that of control group. However, 50 µg/ml baby ginger treatment visibly decreased intracellular lipid accumulation, as indicated by reduced Oil Red O staining. Quantification of lipid staining by measuring absorbance at 490 nm, oil droplet in HepG2 was significantly decreased at (*p* < .05) to 80% in week 1 ginger extract compared with control cells (Figure 5e). In contrast, mature ginger (week 16) extract showed no effect on intracellular lipid formation when compared to that with control treatment at any concentration. This result is well correlated with our findings that baby ginger extract played more regulation and protection roles in fatty acid accumulation compared with the matured ginger extract. Extracts from baby ginger significantly attenuated the overproduction of intracellular lipid droplet and decreased triglyceride contents in HepG2 cells. Gingerol may play an important role in ameliorating and preventing lipid accumulation and metabolism. Tzeng reported that 6‐gingerol inhibited the triglyceride accumulations in HepG2 cells induced by free fatty acids palmitate and oleate mixture (Tzeng et al., 2015). Furthermore, 6‐gingerol significantly attenuated the excessive production of inflammatory cytokines MCP‐1, TNF‐α, and IL‐6. The effects of 6‐gingerol on experimental models of non‐alcoholic fatty liver disease (NAFLD) were also reported. 6‐Gingerol (100 mg kg^−1^ day^−1^) exhibited protective role through regulating key genes related to lipid metabolism and inflammation in male C57BL/6 mice with steatohepatitis induced by a methionine and choline‐deficient (MCD) diet. 6‐gingerol significantly decreased the expressions of inflammatory cytokine genes MCP‐1, TNF‐α, IL‐6, and NF‐κB at both protein and mRNA levels. NAFLD is one of the most predominant forms of chronic liver disease and is commonly linked with metabolic syndrome, such as obesity and type 2 diabetes. Potential therapeutic approaches of 6‐gingerol may involve suppression of oxidative stress and/or pro‐inflammatory responses to prevent against the development of NAFLD. Coincidently, the baby ginger extracts contained highest amount of 6‐gingerol compared with the matured ginger. Based on the evidence in the literature, it is likely that higher levels of 6‐gingerol in baby ginger may have played a role of its superior anti‐oxidation and biological activities. Further research is needed to demonstrate effectiveness of baby ginger in comparison with the mature ginger in an appropriate animal model. Our data strongly suggest that baby ginger can significantly inhibit intracellular lipid accumulation in a dose‐dependent manner.

### 3.6 Ginger extracts attenuate triglyceride content in oleic acid‐treated HepG2 cell

**FIGURE 6:**
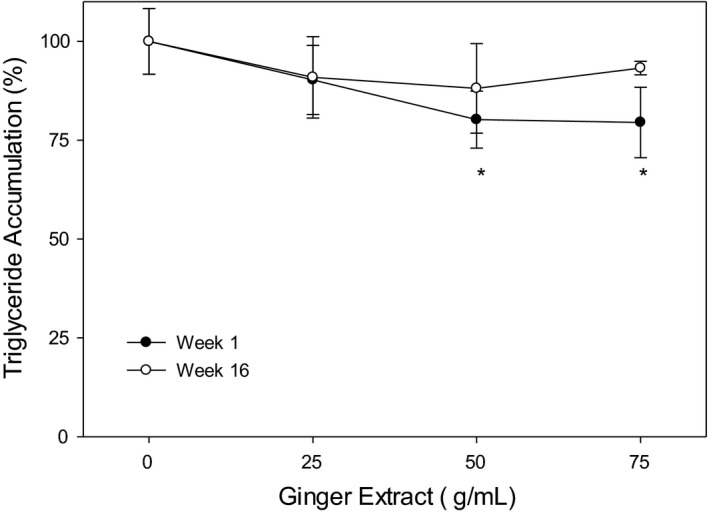
Effects of ginger extracts on inhibiting triglyceride accumulation in HepG2 cells. Triglyceride content was quantified as described in the text. The results represent mean ± *SD* (*n* = 5). *: *p* < .05 compared to 0 g/ml

To further understand the effect of ginger extract on lipid accumulation, we quantified triglyceride in the oleic acid‐treated HepG2 cells. As shown in Figure 6, the triglyceride accumulation was drastically decreased to 79%–81% (*p* < .05) when treated with 50 and 75 µg/ml baby ginger extracts when compared with untreated group. In contrast, no statistical difference was observed, even with 75 µg/ml, on treatment with mature ginger on triglyceride accumulation. These results strongly demonstrate an effect of baby ginger on blocking the triglyceride accumulation in oleic acid‐treated HepG2 cell.

Consumption of dietary ginger products improves lipid metabolism and exhibits positive effects in wide range of biological activities as demonstrated in animal models. Ginger extract was shown as an anti‐hyperlipidemia agent when rats were fed with high‐fat diet. In that study, supplementation with ginger extract reduced final body weight and epididymal adipose tissue mass without affecting energy intake (Oh et al., 2017). Furthermore, ginger has shown to improve serum lipid profiles by lowering TG, TC, and LDL‐C and by increasing HDL‐C in HF diet‐fed rats (Oh et al., 2017). In our current study, to evaluate lipid metabolism, oil droplet accumulation and triglyceride content in human HepG2 hepatic cells were measured to compare effects between baby ginger and matured ginger. At comparable doses, matured ginger extracts did not show significant reduction for both lipid accumulation and triglyceride content, whereas baby ginger extracts significantly inhibited lipid accumulation and attenuated triglyceride content; both were reduced by approximately 20% of the control values. It is possible that a higher concentration of mature ginger extract will induce changes similar to baby ginger. Our observations indicate that baby ginger extracts could have a better potential effect on human being to control body weight and diseases related with lipid accumulation. Further investigation is needed to understand the molecular mechanisms regulating cellular processes and genes expressions involved in hepatic cell adipogenesis and lipolysis.

### 3.7 Ginger extracts inhibit in vitro α‐Amylase activity

**FIGURE 7:**
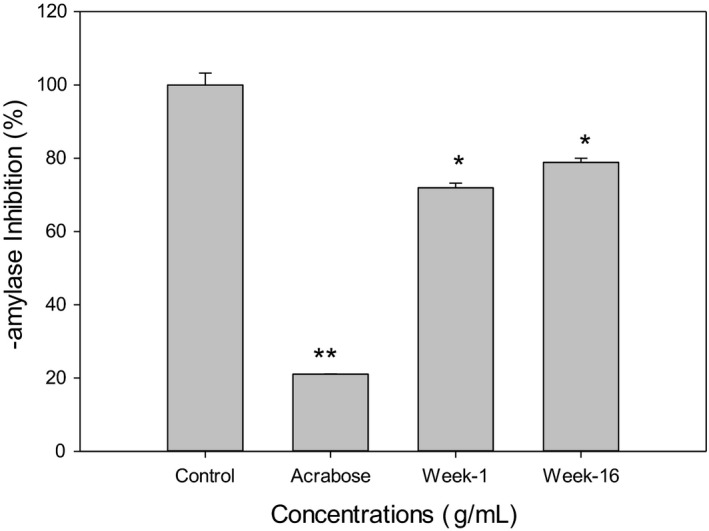
Effect of ginger extract on in vitro α‐amylase inhibition. The porcine pancreatic aamylase was treated with 100 mg/ml each of Acarbose, and ginger extracts. Control was treated with equal amounts of vehicle (PBS). The results represent mean ± *SD* (*n* = 5). *: *p* < .05, **p < .01 compared to control

To evaluate the differences between the baby ginger and the mature ginger on the effects of glucose homeostasis, we examined if ginger extract inhibits α‐amylase activity in vitro and promotes glucose uptake in hepatic cells. Data presented in Figure 7 showed that 0.1mg/ml of methanol extracts of week 1 and week 16 ginger significant inhibited α‐amylase enzyme activity by 28.1% and 21.1%, respectively. Acarbose, which was used as a reference standard to confirm the assay procedure, showed much larger effects on α‐amylase inhibition. There was no significant difference between week 1 and week 16 ginger extracts. Alpha‐amylase is one of the key enzymes that plays a key role in carbohydrate metabolism, by involving in the hydrolysis of α‐bonds of polysaccharides and releasing glucose and maltose (Meisler & Ting, 1993; Perry et al., 2007). Inhibition of this enzyme can delay the carbohydrate digestion and glucose absorption and will ultimately lower postprandial hyperglycemia. Recently, there has been a growing interest in food‐based α‐amylase inhibitors, which provide potential therapeutic approaches in the management of type 2 diabetes and its complication. Rani suggested that ginger ethyl acetate extract inhibited α‐amylase activity (Rani et al., 2011). No significant inhibition of α‐glucosidase enzyme activity was observed by week 1 or week 16 ginger extract treatments (Data not shown).

### 3.8 Ginger extracts ameliorate glucose uptake in HepG2 cell

**FIGURE 8:**
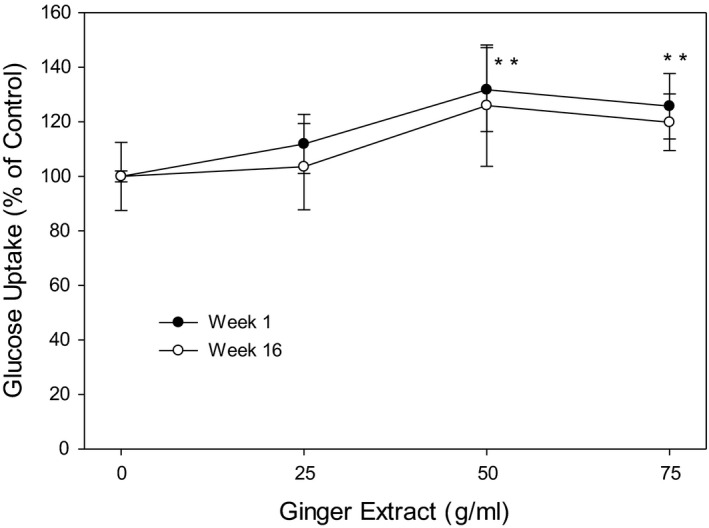
Effects of ginger extracts on glucose uptake in HepG2 cell. HepG2 cells were treated with baby and mature ginger treatments: 0, 25, 50, 75 μg/ml. Uptake of 2‐NBDG was quantified by measuring Fluorescence at 485/650 nm excitation/emission. The results represent mean ± *SD* (*n* = 5). * *p* < .05 compared to 0 g/ml

Glucose uptake by HepG2 cells was significantly increased by 20%–30% on ginger extracts treatment at both 50 and 75 µg/ml; however, no such effect was detected in week 16 ginger samples (Figure 8).

Li demonstrated that ginger extract had the positive effect on stimulating glucose uptake in L6 rat skeletal muscle cells (Li et al., 2012). In a separate experiment, we have also tested the effect of 6‐gingerol on glucose uptake in 3T3L1 cells. Our data indicate that 6‐gingerol caused a dose‐dependent increase in glucose uptake resulting a 50% increase at 15 µg/ml concentration (unpublished data). Considering the data presented in Figure 3, it appears that about 1 g of dried ginger powder could provide a dose in range of 10–15 µg of 6‐gingerol for its biological activities.

Improving glucose uptake into insulin responsive tissues is the fundamental strategy for re‐establishment of glucose homeostasis and battling type 2 diabetes (Krentz & Bailey, 2005). Our results showed that baby ginger extracts inhibited about 30% of yeast α‐amylase activity in vitro and increased glucose uptake in HepG2 cell by 20% more. Mature ginger extract had similar effect on glucose uptake comparing with the baby ginger extract. Our data suggest that baby ginger has a potential on lowering blood glucose levels by inhibition of α‐amylase activity and improving glucose uptake. This finding provides further evidence to support the use of baby ginger in the management of hyperglycemia through control and delaying of postprandial glucose levels in prevention of diabetes.

## 4 CONCLUSIONS

The present study suggested that baby ginger, which was harvested in an early stage, is superior in their total phenolic contents and anti‐oxidation properties compared with the ginger samples harvested at a later stage. Baby ginger has highest key phytochemicals such as 6‐gingerol, 6‐paradol, and 6‐shogaol. Baby ginger extract inhibited fat accumulation and triglyceride contents in oleic acid‐treated HepG2 cells, stimulated glucose uptake in HepG2 cells, and inhibited α‐amylase activity in vitro. This study provides valuable insights into the potential beneficial effects of ginger in controlling obesity and diabetes.

## CONFLICT OF INTEREST

All authors declare no conflict of interest.

## ETHICAL APPROVAL

This study does not involve any human or animal testing.

## Data Availability

The data will be available on request.
